# Supplementary figures and images for: N-Linked Glycosylation Modulates Golgi-Independent Vacuolar Sorting Mediated by the Plant Specific Insert
Source: Plants (Basel). 2019 Aug 30;8(9):312. doi: 10.3390/plants8090312 (PMC6784193; doi:10.3390/plants8090312)

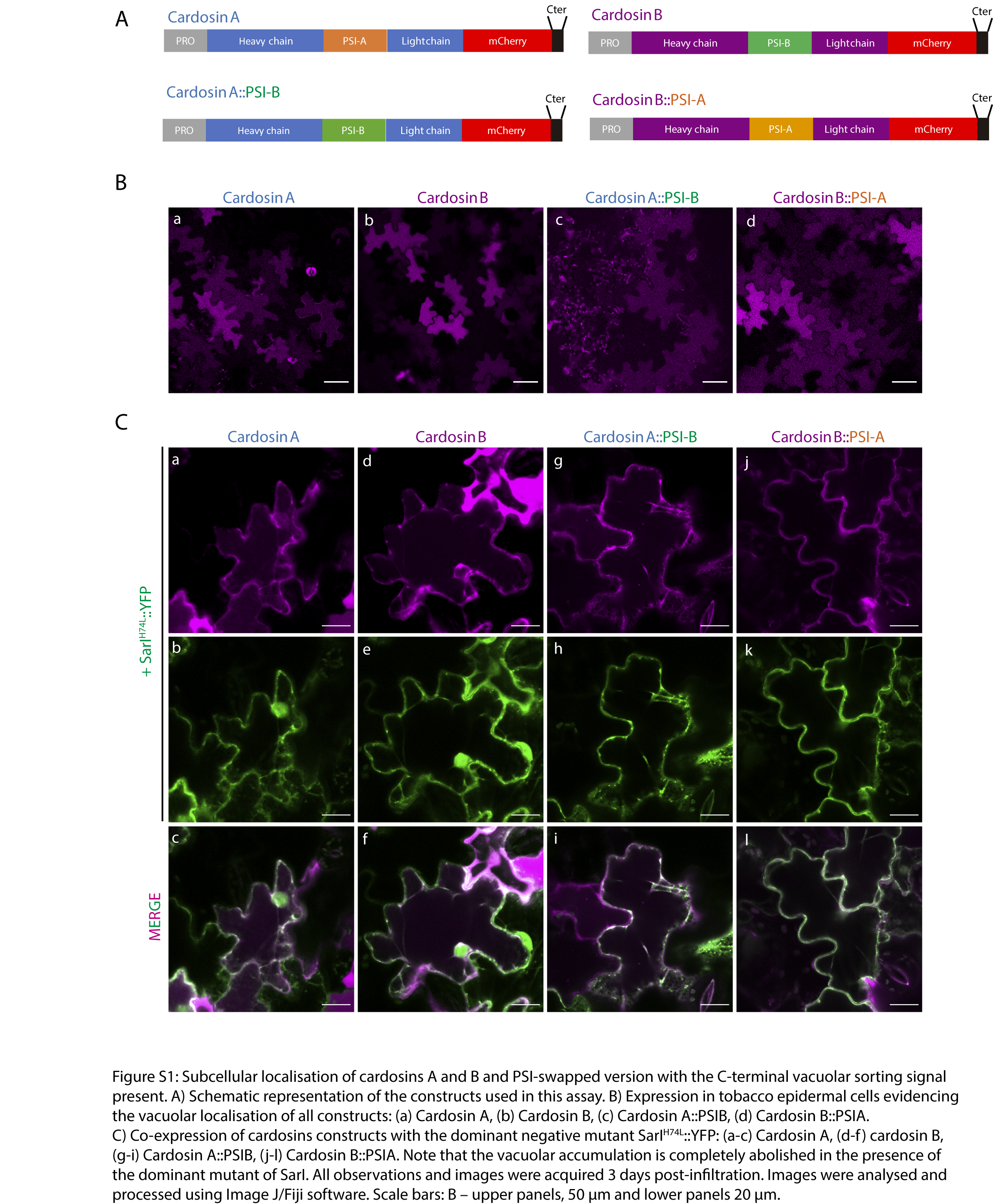

Supplement: Supplementary file 1 [file plants-08-00312-s001.zip › Sup Figures/Sup Figure 1_revised.tif]

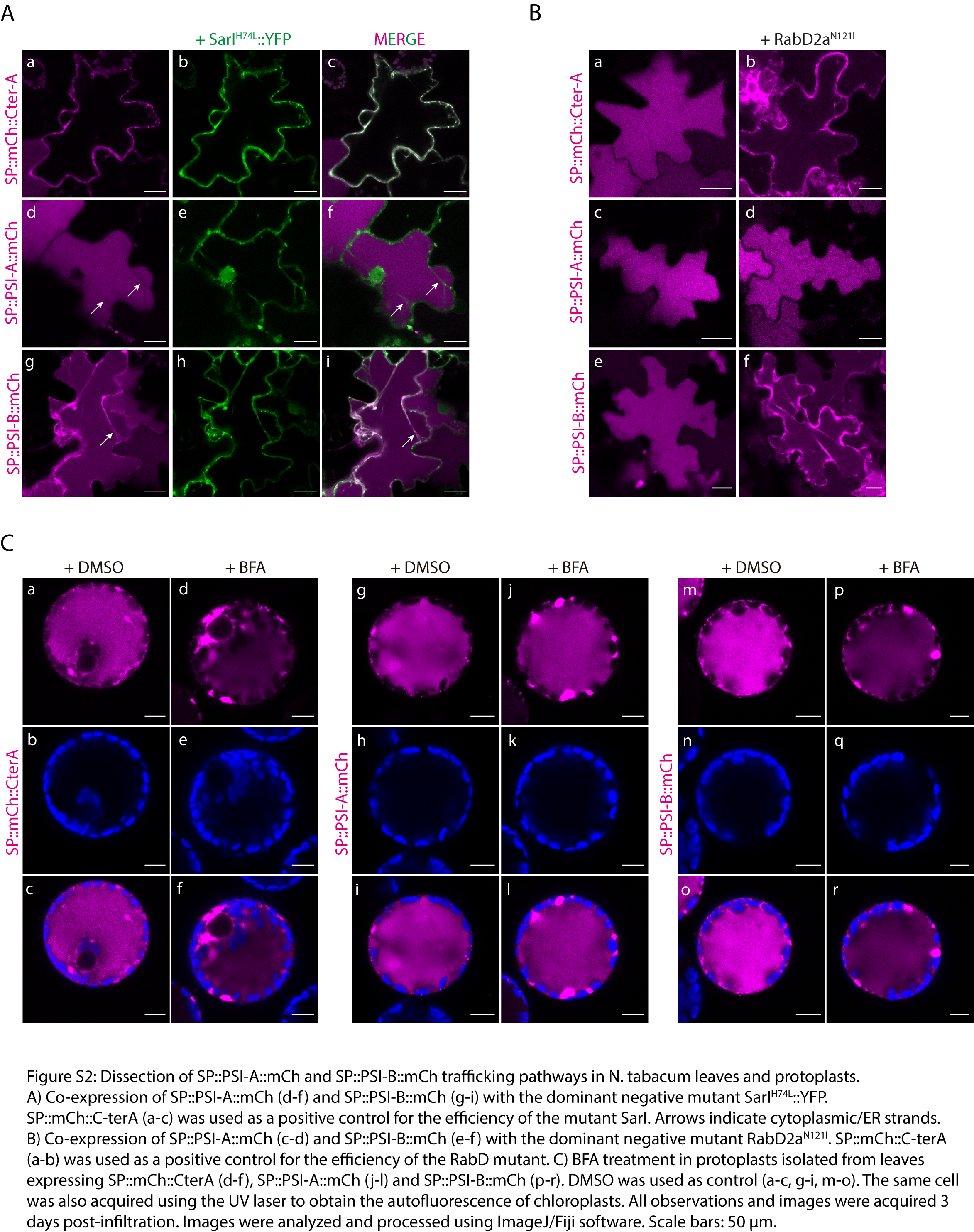

Supplement: Supplementary file 1 [file plants-08-00312-s001.zip › Sup Figures/Sup Figure 2_revised.tif]

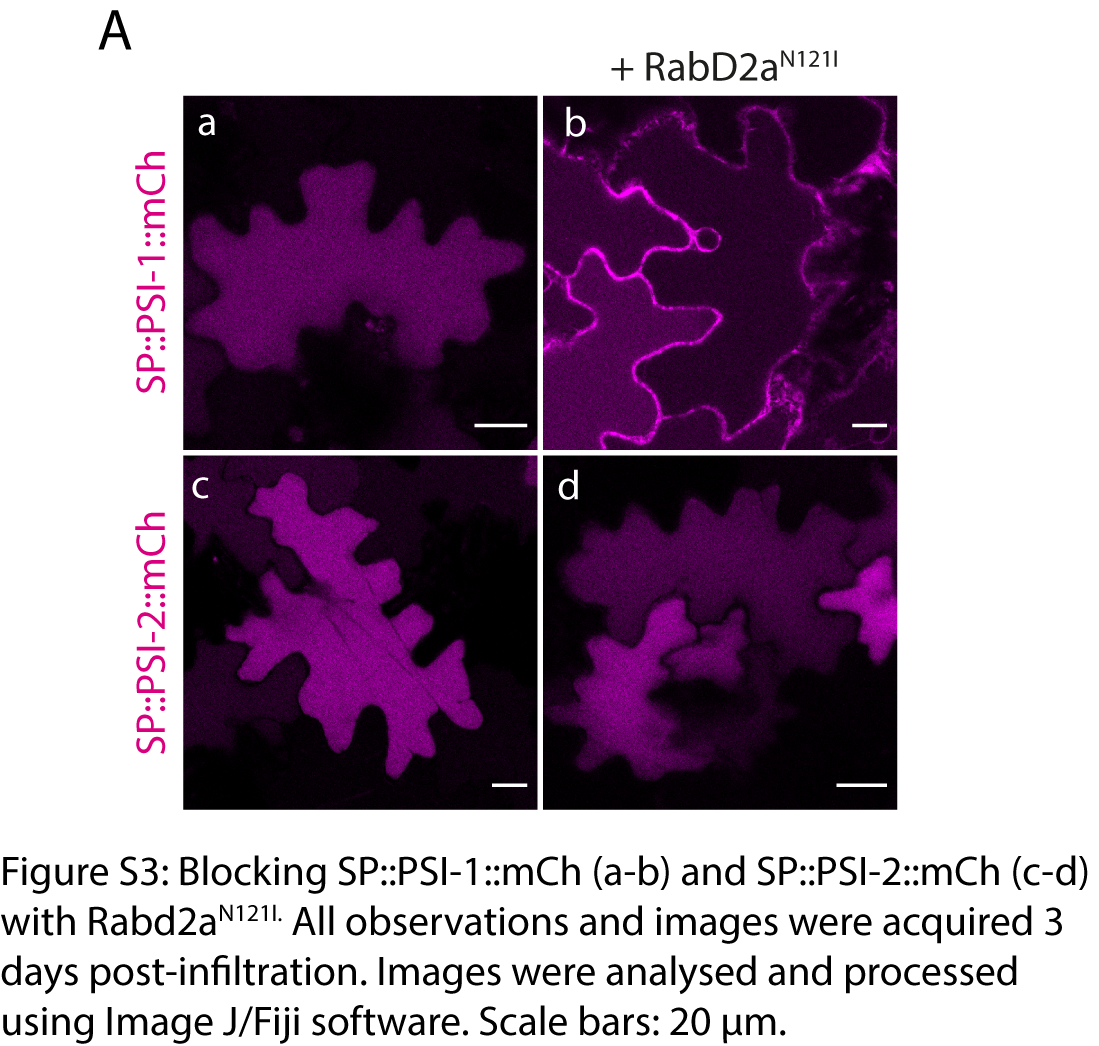

Supplement: Supplementary file 1 [file plants-08-00312-s001.zip › Sup Figures/Sup Figure 3_revised.tif]
